# An effective algorithm to detect the possibility of being MSI phenotype in endometrial cancer given the BMI status and histological subtype: a statistical study

**DOI:** 10.1007/s12094-022-02837-4

**Published:** 2022-05-05

**Authors:** Isabel González Villa, Enrique Francisco González Dávila, Idaira Jael Expósito Afonso, Leynis Isabel Martínez Blanco, Juan Francisco Loro Ferrer, Juan José Cabrera Galván

**Affiliations:** 1 Pathological Anatomy Service, Canary University Hospital, Tenerife, Spain; 2 Faculty of Health Sciences, University of Las Palmas de Gran Canaria, Las Palmas de Gran Canaria, Spain; 3 Department of Mathematics, Statistics and Operations Research, University of La Laguna, Tenerife, Spain; 4 Pathological Anatomy Service, Nuestra Señora de Candelaria University Hospital, Canary Islands Health Service, Tenerife, Spain; 5 Department of Clinical Sciences, Faculty of Health Sciences, University of Las Palmas de Gran Canaria, Las Palmas de Gran Canaria, Spain; 6 Pathological Anatomy Unit, Morphology Department, University Institute for Biomedical and Health Research (IUIBIS), University of Las Palmas de Gran Canaria, Las Palmas de Gran Canaria, Spain

**Keywords:** Endometrial neoplasms, DNA mismatch repair deficiency, MSI phenotype, Immunohistochemistry, Body mass index

## Abstract

**Purpose:**

In endometrial cancer, the incidence of mutations in mismatch repair genes (MMR) is estimated at 17–30%. Patients with alterations at this level (MSI) are known to have different clinical and anatomopathological characteristics than those without this genetic alteration (MSS). In this study, we aim to identify the MSI phenotype in patients who underwent hysterectomy for endometrial cancer. We assessed the correlation of this phenotype with anatomoclinical parameters such as obesity and histological subtype.

**Methods/patients:**

Clinical and anatomopathological data were collected from 147 patients diagnosed with endometrial cancer and an immunohistochemical study of MMR system proteins was performed. PMS2 and MSH6 proteins were evaluated as primary screening and subsequent evaluation of MLH1 and MSH6, respectively, if the former were negative. Statistical association between the anatomopathological data and the immunohistochemical result was analyzed.

**Results and conclusions:**

22.4% of our patients were MSI phenotype. We obtained statistically significant differences by multivariate analysis between endometrioid subtype and higher FIGO classification grade with MSI phenotype and obesity with MSS phenotype. Given these statistical results, we propose a function for predicting the probability of being MSI phenotype taking into account the histological subtype (endometrioid/non-endometrioid carcinoma) and FIGO grade as well as obesity. This prediction may be useful prior to hysterectomy, for genetic study of the MLH1 promoter and subsequent genetic counseling.

## Introduction

Endometrial cancer is the seventh most common in incidence worldwide, being the fourth most common in women in Spain, with 6,874 new diagnoses in 2018, and the fourth most common in incidence in the autonomous community of the Canary Islands, with 298 cases [1]. On the island of Tenerife, 137 new cases were diagnosed that year [2].

In endometrial cancer, the incidence of mutations in mismatch repair genes (MMR) is estimated at 17–30% [3–5]. The proteins of the MMR system are arranged in dimers, MLH1 together with PMS2 and MSH2 together with MSH6. The dominant proteins are MLH1 and MSH2, respectively, while PMS2 and MSH6 are unstable proteins when unpaired. Based on the functional structure of the heterodimer, it is feasible to perform an immunohistochemical panel of PMS2 and MSH6 markers as primary screening for MMR deficiency [6]. The study of the microsatellite instability (MSI) phenotype by these techniques is validated by standardized guidelines that confirm the direct association between MMR genetic study and protein assessment [7–9]. The distinction between patients with MSI phenotype and those with stable phenotype (MSS) allows the identification of patients who are candidates for MMR germline testing and the differentiation of Lynch syndrome cases from sporadic cases, as they present different anatomoclinical, prognostic and therapeutic factors [4].

Despite the scarce literature correlating MSI status and endometrial cancer, several studies have suggested that any histological subtype of endometrial cancer can be MSI, with a distribution similar to that found in the MSS population [4, 10–14].

One of the best known risk factors associated with endometrial cancer is obesity [15]. Thus, women with a higher body mass index (BMI) have been studied to have an increased risk of endometrial cancer with the MSS phenotype, but not with the MSI phenotype. Therefore, a differentiated risk of endometrial cancer associated with BMI based on the MMR phenotype is suggested [15–17].

To our knowledge, there are no statistical algorithms that predict the probability of having MSI phenotype in relation to anatomoclinical factors such as obesity and histological subtype, which motivates the present work.

## Material and methods

We conducted a *prospective observational case study* of all patients diagnosed with endometrial cancer after undergoing total hysterectomy at the Anatomic Pathology Department of the Complejo Hospitalario Universitario de Canarias (Tenerife, Spain) from January 2017 to May 2020 (40 months). The geographical reference area of this center is the northern area and the Isla Baja region of the island of Tenerife with a population census of 187,998 women as of 1 January 2018 [18].

### Inclusion and exclusion criteria

We included in this work patients over 18 years who had undergone total hysterectomy for endometrial cancer from January 2017 to May 2020 in our hospital. Inclusion criteria consisted of a previous endometrial biopsy performed at the hospital center and a clinical history that included data on body mass index, pre-or postmenopausal status, and the presence of previous tumor pathology. Tumor location (uterine cavity or lower uterine segment) in the macroscopic report was also considered an inclusion criterion. Cases that did not meet these criteria were excluded.

### Clinical data

Clinical data were extracted from the center’s electronic record. The variables collected were age at diagnosis (dividing patients into those older and younger than 70 years according to 19, 20), pre- or post-menopausal status, BMI (kg/m^2^), existence of previous tumor pathology, and death during the study as a consequence of such pathology.

BMI (kg/m^2^) was collected according to nutritional status: underweight (if BMI (kg/m^2^) < 18.5), normal weight (18.5–24.9), overweight (25–29.9), obese I (30–34.9), obese II (35–39.9) and obese III (greater than 40) [21]. Also, for statistical reasons, patients were divided into non-obese (BMI (kg/m^2^) < 30) and obese (BMI (kg/m^2^) ≥ 30) [22].

### Pathological aspects

Hysterectomy specimens were fixed in 10% buffered formaldehyde for 24–72 h and subsequently, histological sections were embedded in paraffin. Sections of 3 μm were made and stained with conventional hematoxylin–eosin. The diagnosis was confirmed independently by three subspecialized pathologists in gynecological pathology and any discrepancies between them were resolved by consensus.

The anatomopathological variables analyzed were the macroscopic location of the tumor in the uterine cavity or in the lower uterine segment [23], the histological subtype according to the criteria of the World Health Organization (WHO, 2020 5th Edition), and its classification according to the FIGO (International Federation of Gynecology and Obstetrics) system [24], the presence or absence of lymphovascular invasion, the pathological staging according to the AJCC TNM indications (8th edition) [25] and the presence of associated non-tumor pathology.

### Immunohistochemical study

An immunohistochemical study of DNA repair proteins was performed. Staining was performed on 3 μm thick sections on automated silanized slides. We used four prediluted antibodies (prediluted and incubated for 60 min-each one), from the ROCHE VENTANA immunohistochemistry panel: for MLH1, mouse anti-human monoclonal antibody, clone M1 (1 μg/mL); for PMS2, mouse anti-human monoclonal antibody, clone A16-4 (1 μg/mL); for MSH2, mouse anti-human monoclonal antibody, clone G219-1129 (1 μg/mL); and for MSH6, rabbit anti-human monoclonal antibody, clone SP93 (1 μg/mL).

The presence or absence of nuclear staining was assessed. Corresponding normal tissue (non-tumor epithelial cells as well as lymphocytes and endometrial stromal cells) provided a positive internal control.

Taking into account the immunohistochemical panel for MMR proteins of Hall et al. [6], PMS2 and MSH6 expression is assessed first and, in case of loss of nuclear expression, MLH1 and MSH2 are studied, respectively to differentiate isolated from concomitant loss.

The immunohistochemical variables assessed were expression of intact nuclear protein (MSS phenotype) and loss of nuclear expression of any protein (MSI phenotype) [26, 27].

### Statistical analysis

Statistical analysis was performed using IBM Statistics SPSS V25.0 software, being considered significant when *p* value < 0.05. For the comparison of continuous variables in two groups, the Student *t* test and contingency tables (chi-square test and Fisher’s exact test for 2 × 2 tables) were used for the comparison of categorical variants. A logistic regression model was used for the prediction of MSI (where *p(MSI)* = probability of being MSI) with the backward (Wald) method for variable selection. Initially, the variables age, pre- or post-menopausal status, BMI status (obese or non-obese), pathological staging (pT1, pT2, pT3, and pT4), macroscopic tumor location, and histological type and grade (non-endometrioid, endometrioid grade 1, grade 2 and grade 3) were entered. The area under the ROC curve (AUROC) was calculated. With the selected variables, the linear predictor *η* was constructed, obtaining the estimated probability of being MSI from the formula [28]:

1 $$\widehat{p(MSI)}={e}^{\eta }/(1+{e}^{\eta })$$

where *e* represents the exponential. Data were described by showing the mean ± standard deviation for continuous variables and the frequency (%) for categorical variables.

## Results

The total number of cases under study was 147 patients with a mean age of 64.3 (11.7) years, ranging from 39 to 91 years, with 36.1% of patients being over 70 years of age (Table 1). Of the patients, 82.3% (121/147) were postmenopausal and 6.1% (9/147) had previous tumor pathology (six diagnosed with breast cancer, one with renal cancer, one with thyroid cancer, and one with bladder cancer). During the study period, 10 patients died as a result of endometrial pathology.

**Table 1:** Distribution of the variables collected according to the MMR phenotype

	Total (*N* = 147)	MSI (*N* = 33)	MSS (*N* = 114)	*p* value
Age at diagnosis (years)	64.3 ± 11.7	64.9 ± 11.5	64.1 ± 11.8	0.724
Age at diagnosis				0.838
< 70	94 (63.9)	22 (66.7)	72 (63.2)	
≥ 70	53 (36.1)	11 (33.3)	42 (36.8)	
Post-menopausal status	121 (82.3)	27 (81.8)	94 (82.5)	0.933
Obesity	80 (54.5)	12 (36.4)	68 (59.6)	0.018
Previous tumor pathology	9 (6.1)	4 (12.1)	5 (4.4)	0.115
Death	10 (6.8)	1 (3.0)	9 (7.9)	0.458
Histological subtype				0.023
Non-endometrioid	23 (15.6)	3 (9.1)	20 (17.5)	
Endometrioid	124 (84.3)	30 (90.9)	94 (82.5)	
Grade 1	95 (64.6)	18 (54.5)	77 (67.6)	
Grade 2	18 (12.2)	6 (18.2)	12 (10.5)	
Grade 3	11 (7.4)	6 (18.2)	5 (4.4)	
Stage				0.249
pT1	126 (85.7)	29 (87.9)	97 (85.1)	
pT2	13 (8.8)	2 (6.1)	11 (9.6)	
pT3	7 (4.7)	1 (3.0)	6 (5.3)	
pT4	1 (0.6)	1 (3.0)	-	
Stage				0.538
pT1a	94 (63.9)	23 (69.7)	71 (62.3)	
Rest	53 (36.0)	10 (30.3)	43 (37.7)	
Macroscopic location				0.711
Uterine cavity	136 (92.5)	30 (90.9)	106 (93.0)	
Lower uterine segment	11 (7.4)	3 (9.1)	8 (7.0)	
Lymphovascular invasion	27 (18.4)	8 (24.2)	19 (16.7)	0.318
Associated non-tumor pathology				0.345
Absent	37 (25.1)	6 (18.2)	31 (27.2)	
Atrophy	22 (14.9)	4 (12.1)	18 (15.8)	
Hyperplasia	32 (21.8)	6 (18.2)	26 (22.8)	
Others (polyps, leiomyomas, …)	56 (38.0)	17 (51.5)	39 (34.2)	

According to BMI (kg/m^2^) status, 37 patients were normal weight, 30 were overweight, 32 obese I, 23 obese II, 22 obese III, and 3 obese IV. There were 67 non-obese patients and 80 obese patients (54.5%).

The macroscopic tumor lesion was located in the endometrial cavity in 136 (92.5%) patients and 18.4% had lymphovascular invasion. According to histological type, 124 cases (84.4%) were endometrioid carcinoma, of which 95 were grade 1, 18 grade 2, and 11 grade 3. The 23 patients with non-endometrioid carcinoma were divided into 13 serous carcinomas, 5 carcinosarcomas, 2 mixed carcinomas (one serous, clear cell, and endometrioid; the other serous and clear cell), one mucinous carcinoma, one adenosquamous carcinoma, and one clear cell carcinoma. Stage pT1 was the most frequent with 126 (85.7%) patients (94 cases pT1a and 32 cases pT1b), 13 pT2, 5 pT3a, 2 pT3b, and one pT4. The associated non-tumoral endometrial pathology was varied, with hyperplasia standing out in 32 cases (21.8%).

**Fig. 1:**
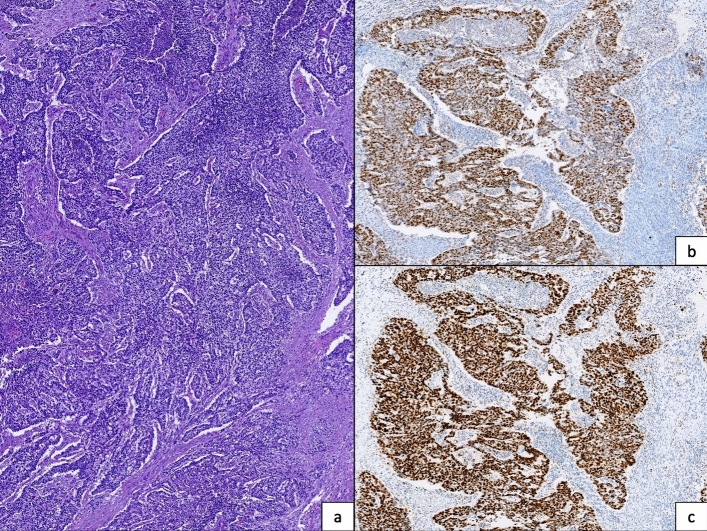
An example of a MSS phenotype case. **a** H&E (× 10): Grade 3 endometrioid carcinoma of endometrium. **b** IHC PMS2: Intact nuclear protein expression. c. IHC MSH6: Intact nuclear protein expression

**Fig. 2:**
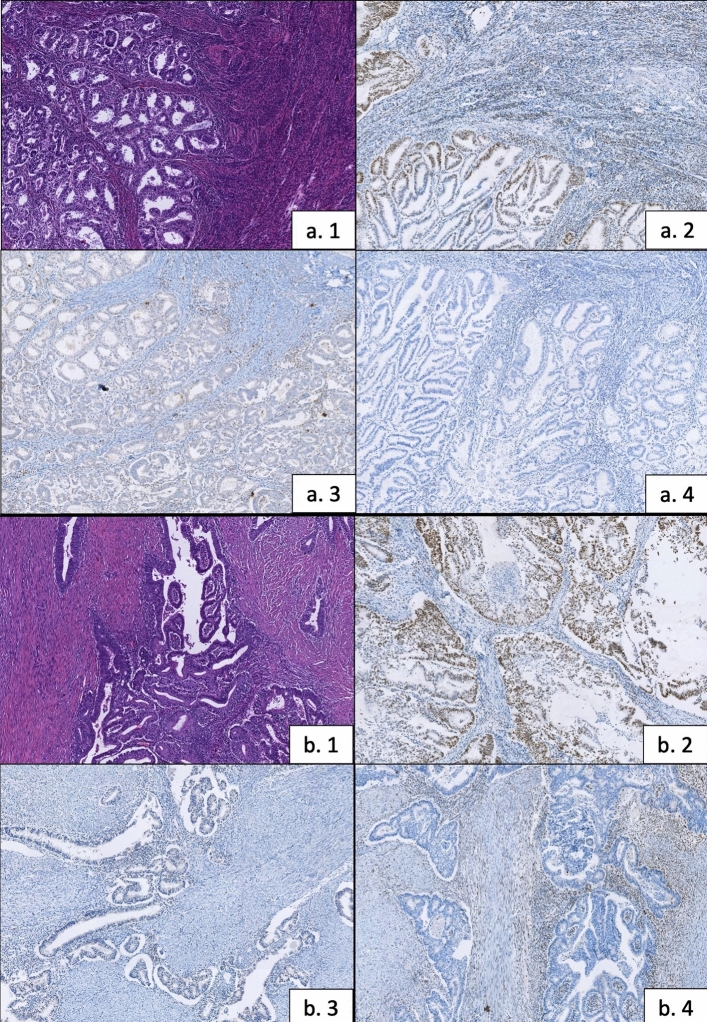
Two examples of MSI phenotype cases. **a. 1** H&E (× 10): Grade 1 endometrioid carcinoma of endometrium. **a. 2** IHC MSH6: Intact nuclear protein expression. **a. 3** IHC PMS2: Loss of protein expression. **a. 4** IHC MLH1: Loss of protein expression. **b. 1** H&E (× 10): Grade 2 endometrioid carcinoma of endometrium. **b. 2** IHC PMS2: Intact nuclear protein expression. **b. 3** IHC MSH6: Loss of protein expression. **b. 4** IHC MSH2: Loss of protein expression

A total of 114 tumors had intact nuclear protein expression, MSS (Fig. 1) and 33 protein loss, MSI (22.4%), of which 25 were due to loss of MLH1 and PMS2 expression, and 8 due to loss of MSH2 and MSH6 expression (Fig. 2). No cases were identified with isolated loss of PMS2 or MSH6 expression.

**Fig. 3:**
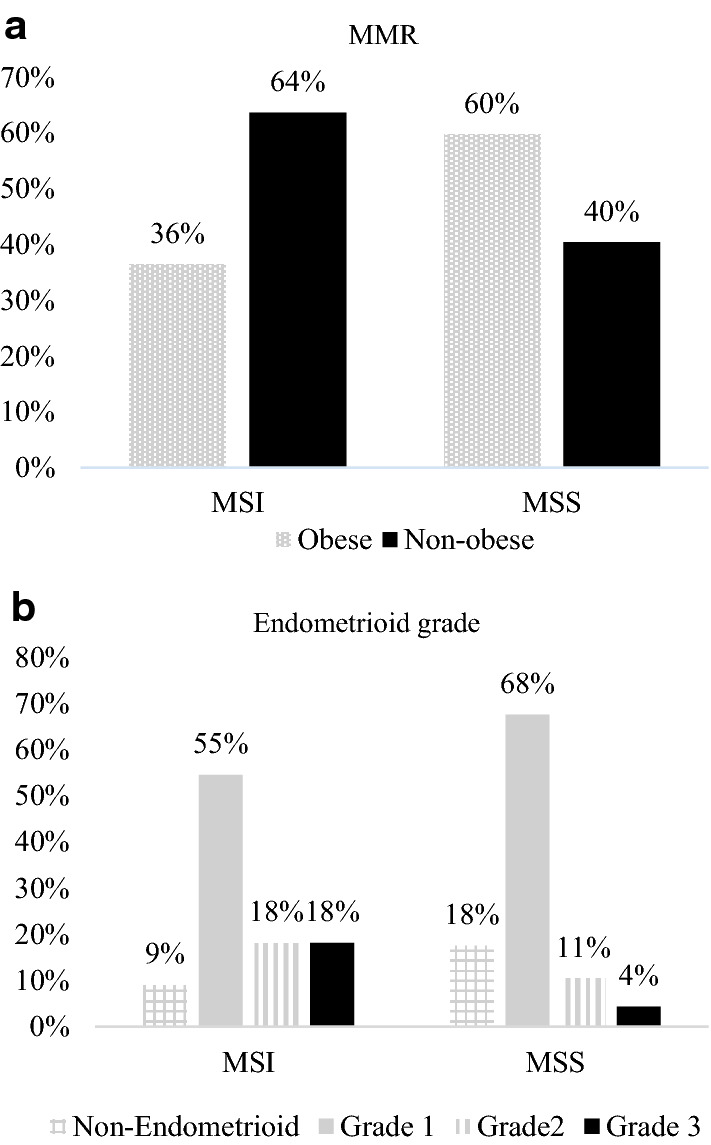
**a** Confronting the MMR phenotype against obesity. 64% of MSI patients are non-obese. While 60% of MSS patients are obese. **b** Confronting the MMR phenotype against the FIGO grade of endometrioid carcinomas. In both cases, most patients present with grade 1 endometrioid carcinoma

No differences were detected in the demographic characteristics or anatomopathological features of the patients based on obesity. Of obese patients, 36% were MSI compared to 64% of non-obese patients (*p* = 0.018) (Fig. 3a). Table 1 shows the data according to the distribution of patients in MSI or MSS. Apart from obesity, there were statistically significant differences in histological type (*p* = 0.023) (Fig. 3b), divided into non-endometrioid and endometrioid subtypes of FIGO grades 1, 2, and 3.

**Fig. 4:**
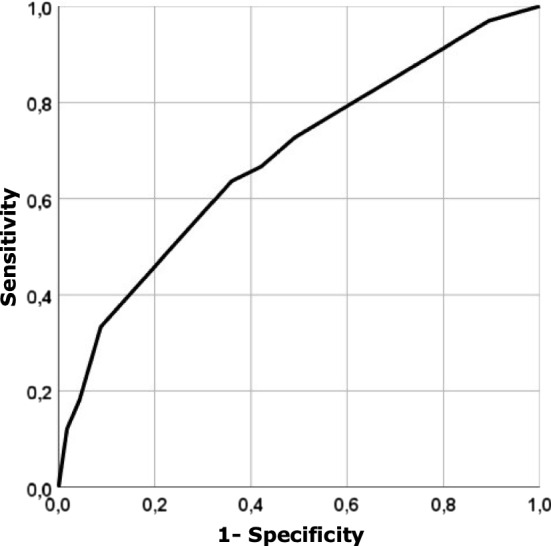
An ROC curve for obesity, histological type (endometrioid- non-endometrioid) and FIGO grade (in endometrioid carcinoma). The area under the ROC curve is 0.683

The variables that remained within the logistic regression model were obesity (*p* = 0.018), and histological type (endometrioid–non-endometrioid) and FIGO grade in endometrioid carcinomas (*p* = 0.023). The logistic regression model (Table 2), indicates that non-obese endometrial cancer patients are 2.487 (95% CI 1.087, 5.690, *p* = 0.031) times more likely to be MSI than obese and that endometrioid grade 3 is 7.866 (95% CI 1.395, 44.370, *p* = 0.019) times more likely to be MSI than non-endometrioid histological type. Overall, both grade 1 and 2 had Odds ratios of 1.583 and 3.109 in favor of MSI over the non-endometrioid group, although these were not significant. The ROC curve is shown in Fig. 4.

**Table 2:** Logistic regression model for the prediction of MSI

	Coefficient	s.e	*p* value	Odds ratio (OR)	IC_95%_ for OR
Non-obese (Ref. Obese)	0.911	0.422	0.031	2.487	(1.087; 5.690)
Endometrioid grade (Ref. Non-endometrioid)			0.048		
Grade 1	0.459	0.681	0.500	1.583	(0.417; 6.008)
Grade 2	1.134	0.808	0.161	3.109	(0.637; 15.160)
Grade 3	2.063	0.883	0.019	7.866	(1.395; 44.370)
Constant	− 2.520	0.699	0.0000	0.080

The area under the ROC curve is 0.683 (95% CI 0.576, 0.790; *p* = 0.001) (Fig. 4). For a false positive rate of 20% it has a sensitivity of 46.1%. From Table 2 we obtain the expression of the linear predictor, *η*, as follows:

$$\eta =-\mathrm{2,369}+\mathrm{0,911}*\left({\text{if}} \;Non-obese\right)+\mathrm{0,459}*\left({\text{if}} \;Endometrioid \,  Grade 1\right)+\mathrm{1,1}*\left({\text{if}} \;Endometrioid \, Grade 2\right)+\mathrm{2,063}* ({\text{if}} \;Endometrioid \, Grade 3)$$

For example, if we have a non-obese woman and endometrioid carcinoma grade 3:

$$\eta =-\mathrm{2,369}+\mathrm{0,911}+\mathrm{2,063}=\mathrm{0,604}$$

So $\widehat{p(MSI)}=\mathrm{0,647}$ , i.e., she would have a 64.7% probability of being MSI. If she were obese with the same degree, this probability would be 42.4%, according to the Eq. (1).

## Discussion

In this study, we identified the MSI phenotype in patients diagnosed with endometrial cancer using immunohistochemical techniques. Following the recommendations of standardized guidelines [7–9], we analyzed its association with clinical variables such as obesity and anatomopathological variables as histological subtypes.

MMR proteins are functionally arranged in heterodimers, MLH1 together with PMS2 and MSH2 together with MSH6. Based on their functional structure, it is possible to perform an immunohistochemical panel of PMS2 and MSH6 markers as primary screening for MMR deficiency [6]. For immunohistochemical assessment we have taken into account the presence or absence of nuclear staining [26, 27, 29], accepting that any positive reaction of tumor cells is considered intact protein expression (MSS phenotype) and that loss of expression, with positive internal control, is considered MSI phenotype. However, there are other ways of assessing this immunohistochemical expression, such as that performed by Barrow et al. [30] using a semi-quantitative study of nuclear staining intensity. In our opinion, the evaluation system we have used is sufficient for the determination of the MSI phenotype, as has already been used [29, 31].

In the present study, 22.4% (33/147) of the patients had MSI phenotypes, which is very similar to other series [4, 15, 17, 26]. The percentage of loss of the MLH1–PMS2 complex was 17% (25/147) while that of the MSH2–MSH6 complex was 5.4% (8/147), with no isolated loss of PMS2 or MSH6 expression identified. This high percentage of MLH1 loss in endometrial cancer can be largely attributed to hypermethylation of the MLH1 gene promoter and not to MMR gene mutations [31]. If we compare our data with those of other series with a similar percentage of MSI phenotype, we can say that loss of the MLH1–PMS2 complex is found in a very similar proportion (15.7% in the series of Joehlin Price et al. [31] and 15.5% in that of Doghri et al. [4]) while the proportion of MSH2–MSH6 loss is somewhat higher (1.9% in the series of Joehlin Price et al. [31] and 2.22% in that of Doghri et al. [4]). We believe that these differences may be due to the fact that in our series we did not identify isolated loss of PMS2 or MSH6, whereas in the series of Joehlin Price et al. [31] the loss of PMS2 is as high as 22%.

In relation to histological subtype, 84.4% were endometrioid carcinomas (124/147), 10.8% were serous (13/147) and the rest (carcinosarcomas, mixed, mucinous, adenosquamous, and clear cell carcinomas) accounted for 4.8% (10/147), with no undifferentiated or undifferentiated carcinomas in our series, figures that coincide with those published by the WHO (2020, 5th Edition). For statistical analysis, we divided the tumors according to histological subtype into endometrioid (84.4%) and non-endometrioid (15.6%). The association between MMR protein deficiency and histological subtype of endometrial cancer is not fully established. Some authors propose that MSI status is more characteristic of endometrioid carcinoma [10–12], while others claim that it is present in both endometrioid carcinomas and non-endometrioid subtypes in the same proportion [13, 31–33]. In our series, 90.9% (30/33) of MSI patients were endometrioid subtype (*p* = 0.023) (Table 1), evidencing the association between MSI status and endometrioid subtype described in other series [10–12].

Apart from the histological subtype, other anatomopathological features associated with MSI status have been considered. Thus, based on studies on the MSI status of colon cancer, Bartosch et al. [14] established several variables to identify the anatomopathological aspects of MSI endometrial cancer: intense immune response manifested as peritumoral infiltration and infiltrating lymphocytes in the tumor, mucinous differentiation, morphological heterogeneity, and location in the lower uterine segment [23], as well as being associated with higher grade, presence of lymphovascular invasion and higher stage.

In our study, we found no statistically significant differences in terms of location in the lower uterine segment, higher stage, or the presence of lymphovascular invasion. However, when we evaluated differences in FIGO grade classification in endometrioid carcinomas, we found differences between those with the MSI phenotype versus the MSS phenotype. There was a predominance of FIGO grade 3 in endometrioid carcinomas with the MSI phenotype compared to those with the MSS phenotype (Table 2).

Regarding the presence of other pathologies in the histological study, Shia et al. [7] found statistically significant differences between the presence of endometrial hyperplasia in MSI cases compared to MSS. In our case, we assessed the presence of hyperplasia, atrophy, and other non-neoplastic endometrial pathologies (polyps, leiomyomas, endometriosis) in both groups without finding statistical significance.

Obesity is one of the best-known risk factors for endometrial cancer [15]. For its measurement, the most accessible and widely used variable has been BMI (kg/m^2^) [15–17]. The percentage of obese women in Spain (BMI (kg/m^2^) ≥ 30) is 16.6% of the population, but in the autonomous community of the Canary Islands, it is 18.8% [34]. We divided the patients according to BMI (kg/m^2^) status, with 45.5% being non-obese patients and 54.5% obese patients, a much higher percentage than in the general population. In this study (Table 1), almost 60% of the MSS patients were obese (BMI (kg/m^2^) ≥ 30) (*p* = 0.018). If we compare the results with the literature, we observe that they are similar to studies by McCourt et al. [16], Cohn et al. [17], and Joehlin-Price et al. [26] agreeing that the higher the BMI (kg/m^2^), the higher the risk of MSS endometrial cancer. While in the series by Amankwa et al. [15], they found that patients with BMI (kg/m^2^) ≥ 30 had twice the risk of being MSI than MSS.

As reflected in the results from our multivariate study, the variables that remained within the logistic regression model were obesity, histological subtype along with FIGO classification grade, and MSI phenotype.

Accordingly (Table 2), we observed that endometrial cancer patients with BMI (kg/m^2^) < 30 are 2.4 times more likely to be MSI than those with BMI (kg/m^2^) ≥ 30 while those with a diagnosis of FIGO grade 3 endometrioid carcinoma are 7.8 times more likely to be MSI than those with a diagnosis of non-endometrioid carcinoma.

This study demonstrates that by applying the predictive equation and as reflected in our results, we can obtain an estimated probability that a patient, given her histological subtype (endometrioid or non-endometrioid) and FIGO grade, as well as her BMI (kg/m^2^) (< 30 or ≥ 30), is an MSI phenotype.

In conclusion, and for clinical practice, both variables, obesity in the clinical history and the histological subtype through previous biopsy, would allow us to determine the probability that the patient is of MSI phenotype, data that would be subsequently confirmed in the hysterectomy specimen with the study of MMR proteins, as recommended by the NCCN [29], except in very specific cases. This information can be of great use when planning a genetic study of the MLH1 promoter and subsequent genetic counseling.

## Data Availability

Not applicable.
